# Characterization of *Aldh2*^*-/-*^ mice as an age-related model of cognitive impairment and Alzheimer’s disease

**DOI:** 10.1186/s13041-015-0117-y

**Published:** 2015-04-25

**Authors:** Yohan D’Souza, Ahmed Elharram, Raquel Soon-Shiong, R David Andrew, Brian M Bennett

**Affiliations:** Department of Biomedical & Molecular Sciences, and Centre for Neuroscience Studies, Faculty of Health Sciences, Queen’s University, Kingston, Ontario, K7L 3N6 Canada

**Keywords:** Alzheimer’s disease, Aldehyde dehydrogenase 2, 4-hydroxynonenal, Oxidative stress, AD mouse model, Cognitive deficits, Synaptic function

## Abstract

**Background:**

The study of late-onset/age-related Alzheimer’s disease (AD)(sporadic AD, 95% of AD cases) has been hampered by a paucity of animal models. Oxidative stress is considered a causative factor in late onset/age-related AD, and aldehyde dehydrogenase 2 (ALDH2) is important for the catabolism of toxic aldehydes associated with oxidative stress. One such toxic aldehyde, the lipid peroxidation product 4-hydroxynonenal (HNE), accumulates in AD brain and is associated with AD pathology. Given this linkage, we hypothesized that in mice lacking ALDH2, there would be increases in HNE and the appearance of AD-like pathological changes.

**Results:**

Changes in relevant AD markers in *Aldh2*^*-/-*^ mice and their wildtype littermates were assessed over a 1 year period. Marked increases in HNE adducts arise in hippocampi from *Aldh2*^*-/-*^ mice, as well as age-related increases in amyloid-beta, p-tau, and activated caspases. Also observed were age-related decreases in pGSK3β, PSD95, synaptophysin, CREB and pCREB. Age-related memory deficits in the novel object recognition and Y maze tasks begin at 3.5-4 months and are maximal at 6.5-7 months. There was decreased performance in the Morris Water Maze task in 6 month old *Aldh2*^*-/-*^ mice. These mice exhibited endothelial dysfunction, increased amyloid-beta in cerebral microvessels, decreases in carbachol-induced pCREB and pERK formation in hippocampal slices, and brain atrophy. These AD-associated pathological changes are rarely observed as a constellation in current AD animal models.

**Conclusions:**

We believe that this new model of age-related cognitive impairment will provide new insight into the pathogenesis and molecular/cellular mechanisms driving neurodegenerative diseases of aging such as AD, and will prove useful for assessing the efficacy of therapeutic agents for improving memory and for slowing, preventing, or reversing AD progression.

**Electronic supplementary material:**

The online version of this article (doi:10.1186/s13041-015-0117-y) contains supplementary material, which is available to authorized users.

## Background

Alzheimer’s disease (AD) is characterized by intraneuronal and extracellular accumulation of amyloid-β peptide (Aβ), intracellular neurofibrillary tangles (NFTs), the major component of which is hyperphosphorylated tau protein (p-tau), disruption of both excitatory amino acid and cholinergic neurotransmission, and loss of vulnerable neurons, notably forebrain cholinergic neurons that project to the cerebral cortex and hippocampus. The search for critically needed AD therapeutics has been dominated by use of a variety of transgenic mouse models of AD, which exhibit neuropathological changes dependent on the overexpression of mutant human genes linked to early-onset, familial AD. However, genetic aberrations account for only a small proportion of AD cases (<5%). The models exhibit Aβ pathology, but rarely together with neuronal loss and aberrant tau phosphorylation. In contrast, the study of late-onset/age-related AD (sporadic AD, >95% of AD cases) has been hampered by a paucity of animal models that mirror *age-related* progression of AD pathologies. Animal models that develop pathology because the animals age, rather than because the pathology is genetically programmed, would be a valuable addition to currently available transgenic models and would be helpful in assessing intervention strategies that slow or reverse the underlying disease process in addition to relieving symptoms.

The early appearance of oxidative stress markers in AD patients and in animal models of AD that *precedes* the onset of cognitive decline and appearance of Aβ plaques and NFTs [1-5], suggests that oxidative damage may be a primary driving force in AD pathogenesis, especially synaptic dysfunction. This forms the basis of the oxidative stress hypothesis of AD [6-8]. 4-hydroxynonenal (HNE) is an important lipid peroxidation product formed during periods of oxidative stress. Both free HNE and HNE protein adducts accumulate in the brains of AD patients [9-16] and in certain transgenic mouse models of AD [5,17]. Many of these proteins have been identified, and oxidative modifications by HNE adduct formation often results in altered activity [reviewed in 18]. These include proteins involved in the regulation of energy metabolism, antioxidant defense, neuronal communication, stress responses, cytoskeletal integrity and cell signaling. This provides the basis for mechanisms of oxidative stress-induced damage that complement or contribute to Aβ-mediated toxicity. HNE also alters the function and disposition of Aβ, through changes in Aβ formation, aggregation, catabolism and clearance [19-27].

The enzyme aldehyde dehydrogenase 2 (ALDH2) is important for the detoxification of endogenous aldehydes such as HNE [28,29], and inhibition of ALDH2 increases vulnerability to HNE-induced damage [30]. ALDH2 is expressed in the frontal and temporal cortex, hippocampus, mid-brain, basal ganglia and cerebellum, primarily in glial cells and neuropil [28]. Importantly, ALDH2 has been localized to reactive glia within senile plaques in the cerebral cortex and hippocampus, and its expression/activity is increased in the temporal cortex and putamen in AD brains [28,31].

Cross-sectional studies have examined the association of AD risk for individuals possessing the Glu504Lys loss of function mutation of ALDH2 (present in 30-50% of the East Asian population). Although meta-analysis of these studies indicated no increased risk of AD associated with the variant ALDH2, subgroup analysis did indicate that the association was significant for males [32]. In one of the few animal studies reported, Ohsawa et al. [33] found that introduction of this mutation into mice resulted in increased HNE formation associated with age-dependent neurodegeneration and memory loss*.* Taken together these data suggest that not only is the ALDH2 pathway critical for the detoxification of HNE in the brain, but also that levels of toxic aldehydes derived from oxidative stress are sufficient to cause neuronal loss and cognitive impairment.

Given the potential importance of HNE in AD pathogenesis and Aβ disposition, we hypothesized that genetic manipulations that increase HNE levels would result in biochemical, histopathological, and cognitive changes that mirror those found in AD. This was assessed using *Aldh2*^*-/-*^ mice as the model.

## Results

### Behavioural analyses

**Figure 1:**
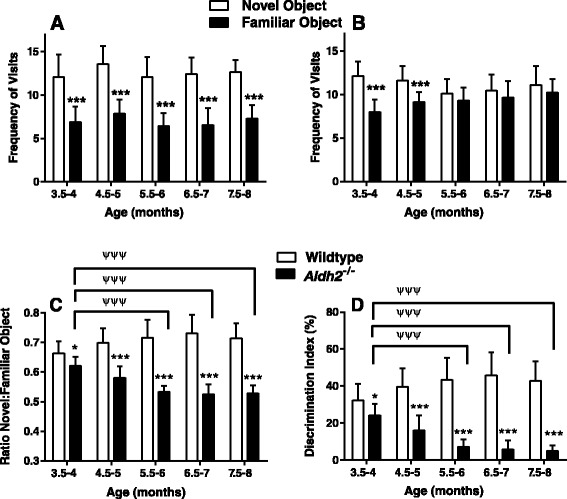
Age-dependent, progressive decline in the Novel Object Recognition (NOR) task performance in *Aldh2* ^*-/-*^ mice. Male and female mice were subjected to the NOR task once per month beginning at three months of age, and the frequency of visits to the objects and the time spent exploring each object was recorded. **A and B**. Frequency of visits to the novel and familiar objects for wildtype mice **(A)** and *Aldh2* ^*-/-*^ mice **(B)**. **(C)**. Ratio of time spent with the novel object in relation to the familiar object. **(D)**. Discrimination index. Data are presented as the mean ± SD (wildtype n = 18, *Aldh2* ^*-/-*^ n = 17) and were analyzed by two-way ANOVA with a Bonferroni post-hoc test. In **A** and **B**, ***, significant difference to the novel object (p < 0.001). In **C** and **D**, *, significant difference from wildtype as indicated (*p < 0.05, **, P < 0.01, ***p < 0.001); ^ψψψ^, significant difference from 3.5-4 month old *Aldh2* ^*-/-*^ mice (p < 0.001).

**Figure 2:**
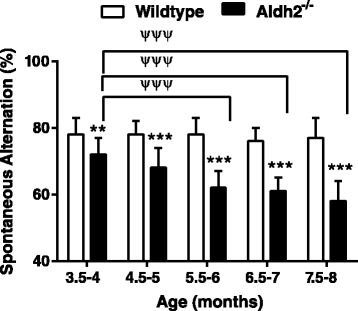
Age-dependent, progressive decline in Y-maze task performance in *Aldh2* ^*-/-*^ mice. The spontaneous alternation rate in the Y-maze task was assessed in male and female mice once per month beginning at three months of age. Data are presented as the mean ± SD (wildtype n = 18, *Aldh2* ^*-/-*^ n = 17) and were analyzed by two-way ANOVA with a Bonferroni post-hoc test. *, significant difference from wildtype as indicated (*p < 0.05, **, p < 0.01, ***p < 0.001); ^ψψψ^, significant difference from 3.5-4 month old *Aldh2* ^*-/-*^ mice (p < 0.001).

**Figure 3:**
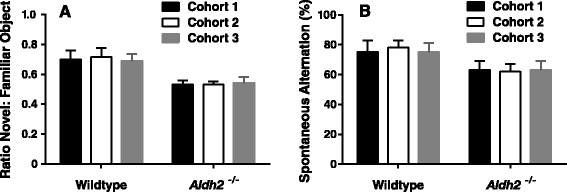
Consistency of NOR and Y-maze tasks in successive cohorts of wildtype and *Aldh2* ^*-/-*^ mice. Three generations of 5.5-6 month old wildtype and *Aldh2* ^*-/-*^ mice were tested in the NOR **(A)** and Y-maze **(B)** tasks. Data are presented as the mean ± SD (wildtype n = 8-18, *Aldh2* ^*-/-*^ n = 11-17) and were analyzed by two-way ANOVA. There were no differences between cohorts of either wildtype or *Aldh2* ^*-/-*^ mice for either behavioural test.

**Figure 4:**
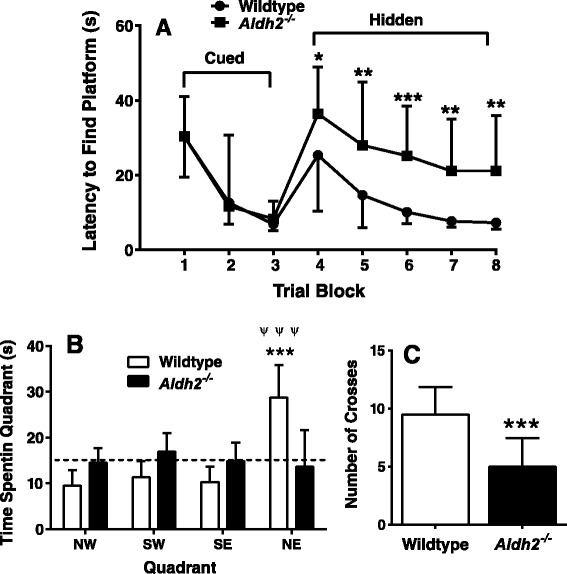
Six month old *Aldh2* ^*-/-*^ mice exhibit decreased performance in the Morris Water Maze task. **(A).** Escape latency (time to reach the hidden platform) was determined in a 3 day cued trial block (4 trials per day) followed by a 5 day hidden trial block (6 trials per day). Day 9 was a probe trial, in which the time spent in the target quadrant (NE)**(B)** and number of platform crosses **(C)** were determined (total time of the trial was 60 s). In **(A)**, data are expressed as the mean ± SD of the average scores in each trial block (n = 18). In **B** and **C**, data are expressed as the mean ± SD (n = 18). In **B**, the dotted line represents the expected time spent in each quadrant by chance alone. Data were analyzed by 2-way ANOVA with a Bonferroni post-hoc test in **A** and **B**, and by a Student’s *t*-test for unpaired data in **C**. In **A** and **C**, *, significant difference from wildtype as indicated (*p < 0.05, ** p < 0.01, ***p < 0.001). In **B**, ***, significant difference from wildtype in the other quadrants; (p < 0.001); ^ψψψ^, significant difference from *Aldh2* ^*-/-*^ in the NE quadrant (*p* < 0.001).

Using the open field novel object recognition (NOR) test and spontaneous alternations in the Y-maze, *Aldh2*^*-/-*^ mice showed a progressive decrease in performance in both memory tasks compared to no change in performance in their wildtype littermates (Figures 1 and 2). Memory deficits began at 3.5-4 months of age and reached a plateau in 6.5 to 7 month old animals. All data were initially tested using a three-way analysis of variance in order to determine if there were sex differences in any of the memory tasks, and this not being the case, data from male and female mice were combined. We also assessed the reproducibility of performance measures between three consecutive generations of 5.5-6 month old animals (Figure 3) and noted very little variation between cohorts. In the Morris Water Maze (MWM) task, there was a significant difference in escape latency for *Aldh2*^*-/-*^ mice compared to wildtype mice in all trial blocks in which the platform was hidden (Figure 4A, trial blocks 4-8). One might not have expected a difference in performance in the first block of hidden platform training, since this was the first instance of the animals having to use spatial cues to find the hidden platform. However, in the first trial of the six trials in this block, there was no difference in escape latency between wildtype and *Aldh2*^*-/-*^ mice (37.3 ± 21.1 s and 41.1 ± 22.1 s, respectively, p > 0.05, Students *t*-test for unpaired data), indicating that the basis for the better overall performance of the wildtype mice in the first block was due shorter escape latencies in the latter trials of the block. In the probe trial, *Aldh2*^*-/-*^ mice spent less time in the target quadrant and had fewer platform crosses compared to wildtype mice (Figure 4B, C). Indeed, *Aldh2*^*-/-*^ mice spent essentially the same amount of time in each quadrant, whereas wildtype mice spent about three times more time in the target quadrant than the other quadrants. Differences in locomotor activity and coordination, assessed using the balance beam task (Additional file 1: Figure S1), as well as behavioural phenotype assessment using the SHIRPA standardized battery (Tables 1 and 2) [34] were not observed in either 2-3 month old or 5-6 month old wildtype and *Aldh2*^*-/-*^ mice, suggesting that diminished cognitive performance was the result of impaired memory and not due to confounding impairments in motor function. This was reinforced by the finding of no difference in latency times in the MWM task during the 3 day cued platform training, in which mice swim to a visible platform (and also suggests no differences in eyesight, swim speed, basic strategies and motivation between wildtype and *Aldh2*^*-/-*^ mice). Also, we did not observe nonspecific behavioural changes such as floating or thygmotaxis in the *Aldh2*^*-/-*^ mice.

**Table 1:** **SHIRPA standardized battery for 2-3 month wildtype and** ***Aldh2*** ^***-/-***^ **mice**

**Test**	**Wildtype (n = 13)**	***Aldh2*** ^***-/-***^ **(n = 20)**
**Viewing jar**		
Body position	Sitting or Standing: 13 (100%)	Sitting or Standing: 20 (100%)
Spontaneous activity	Casual Scratch, Slow Movement 13 (100%)	Casual Scratch, Slow Movement 20 (100%)
Respiration rate	Normal: 13 (100%)	Normal: 20 (100%)
Body tremor	Normal: 13 (100%)	Normal: 20 (100%)
Palpebral closure	Open: 13 (100%)	Open: 20 (100%)
Piloerection	Normal: 13 (100%)	Normal: 20 (100%)
Gait	Normal: 13 (100%)	Normal: 20 (100%)
Pelvic elevation	Normal: 13 (100%)	Normal: 20 (100%)
Tail elevation	Horizontal: 13 (100%)	Horizontal: 20 (100%)
Touch escape	Mild 4 (31%)	Mild 7 (35%)
	Moderate 9 (69%)	Moderate 13 (65%)
Positional passivity	Struggles when held tail: 13 (100%	Struggles when held tail: 20 (100%)
Trunk curl	Present: 13 (100%)	Present: 20 (100%)
Limb grasping	Present: 13 (100%)	Present: 20 (100%)
Grip strength	Active Grip, Effective: 13 (100%)	Active Grip, Effective: 20 (100%)
Body tone	Slight resistance: 13 (100%)	Slight resistance: 20 (100%)
Pinna reflex	Active Retraction: 13 (100%)	Active Retraction: 20 (100%)
**Wire maneuver**		
	Difficulty to Grasp with Hindlegs: 2 (15%)	Difficulty to Grasp with Hindlegs: 4 (20%)
	Active Grip with Hind Legs: 11(85%)	Active Grip with Hind Legs: 16 (80%)
Skin color	Pink: 13(100%)	Pink: 20(100%)
Abdominal tone	Slight resistance: 13 (100%)	Slight resistance: 20 (100%)
Lacrimation	None 13 (100%)	None 20(100%)
Provoked biting	Present 13 (100%)	Present 20(100%)
**Negative geotaxis**	Turns and Climbs Grid 13(100%)	Turns and Climbs Grid 20(100%)
**Handling behaviour**		
Irritability	Present 13(100%)	Present 20 (100%)
Aggression	Present 13 (100%)	Present 20 (100%)
**Visual and auditory proficiency**		
Visual placing test	Before Vibrasse Contact (Visual): 13 (100%)	Before Vibrasse Contact (Visual): 20 (100%)
Preyer’s reflex test	Positive Response to Clap: 13 (100%)	Positive Response to Clap: 20 (100%)

**Table 2:** **SHIRPA standardized battery for 6-7 month wildtype and** ***Aldh2*** ^***-/-***^ **mice**

**Test**	**Wildtype (n = 15)**	***Aldh2*** ^***-/-***^ **(n = 19)**
**Viewing jar**		
Body position	Sitting or Standing: 15 (100%)	Sitting or Standing: 19 (100%)
Spontaneous activity	Casual Scratch, Slow Movement 15 (100%)	Casual Scratch, Slow Movement 19 (100%)
Respiration rate	Normal: 15 (100%)	Normal: 19 (100%)
Body tremor	Normal 15 (100%)	Normal: 19 (100%)
Palpebral closure	Open: 15 (100%)	Open: 19 (100%)
Piloerection	Normal: 15 (100%)	Normal: 19 (100%)
Gait	Normal: 15 (100%)	Normal: 19 (100%)
Pelvic elevation	Normal: 15 (100%)	Normal: 19 (100%)
Tail elevation	Horizontal: 15 (100%)	Horizontal: 19 (100%)
Touch escape	Mild 5 (33%)	Mild 8 (42%)
	Moderate 10 (67%)	Moderate 11 (58%)
Positional passivity	Struggles when held tail: 15 (100%	Struggles when held tail: 19 (100%)
Trunk curl	Present: 15 (100%)	Present: 19 (100%)
Limb grasping	Present: 15 (100%)	Present: 19 (100%)
Grip strength	Active Grip, Effective: 15 (100%)	Active Grip, Effective: 19 (100%)
Body tone	Slight resistance: 15 (100%)	Slight resistance: 19 (100%)
Pinna reflex	Active Retraction: 15 (100%)	Active Retraction: 19 (100%)
**Wire maneuver**		
	Difficulty to Grasp with Hindlegs: 4 (27%)	Difficulty to Grasp with Hindlegs: 5(26%)
	Active Grip with Hind Legs: 11 (73%)	Active Grip with Hind Legs: 14 (74%)
Skin color	Pink: 15(100%)	Pink: 19 (100%)
Abdominal tone	Slight resistance: 15 (100%)	Slight resistance: 19(100%)
Lacrimation	None 15 (100%)	None 19 (100%)
Provoked biting	Present 15 (100%)	Present 19 (100%)
**Negative geotaxis**	Turns and Climbs Grid 15 (100%)	Turns and Climbs Grid 19 (100%)
**Handling behaviour**		
Irritability	Present 15 (100%)	Present 19 (100%)
Aggression	Present 15 (100%)	Present 19(100%)
**Visual and auditory proficiency**		
Visual placing test	Before Vibrasse Contact (Visual): 15 (100%)	Before Vibrasse Contact (Visual): 19 (100%)
Preyer’s reflex test	Positive Response to Clap: 15 (100%)	Positive Response to Clap: 19 (100%)

### Immunoblot analysis of AD markers

**Figure 5:**
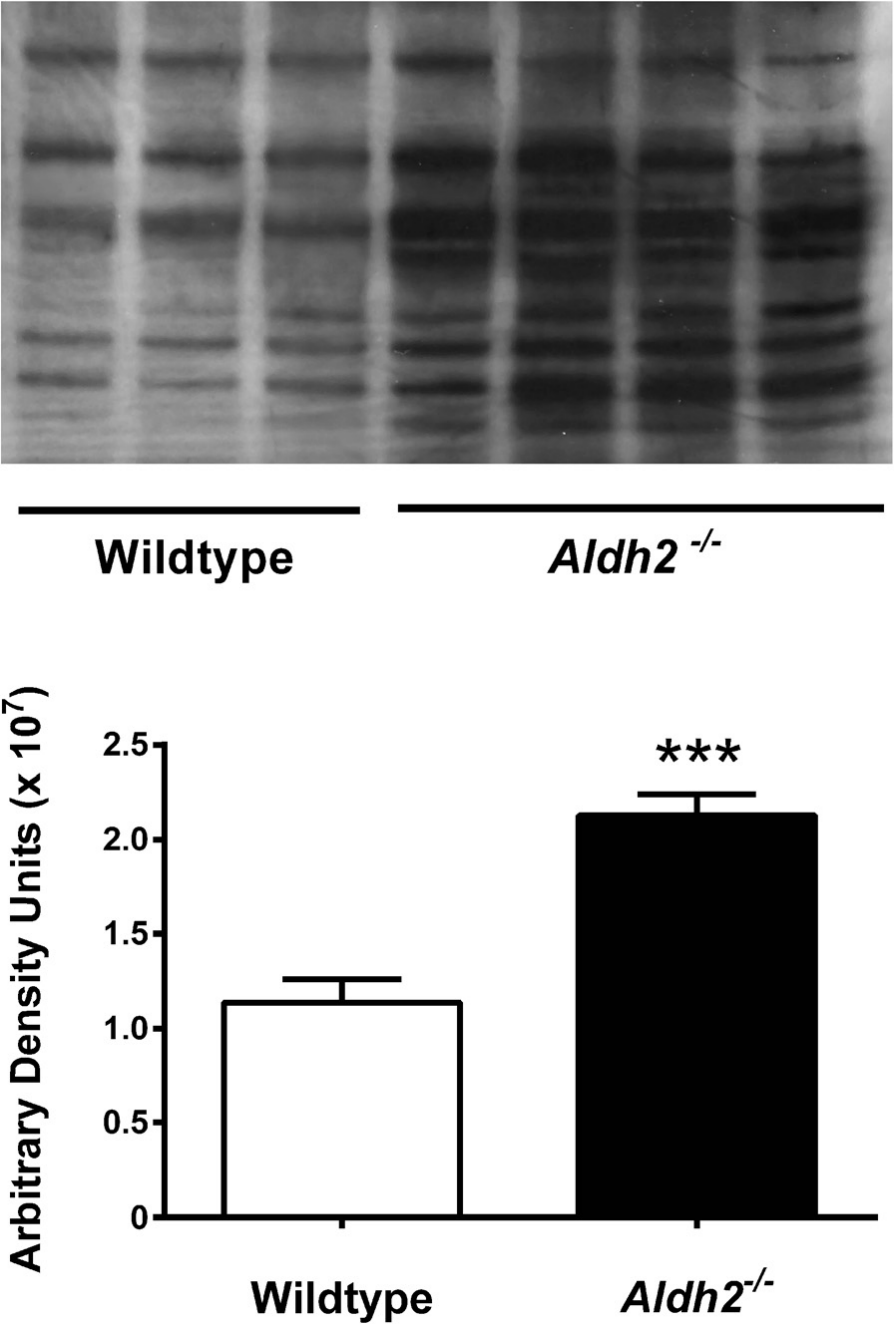
Increased HNE adduct formation in hippocampi from *Aldh2* ^*-/-*^ mice. Immunoblot analysis of hippocampal homogenates (30 μg protein) from 9 month old wildtype or *Aldh2* ^*-/-*^ mice. Proteins were resolved on a 10% SDS-PAGE gel under non-reducing conditions. Immunoreactive bands were quantitated by densitometry. Data are presented as the mean ± SD (n = 3-4) and were analyzed by a Student’s *t*-test for unpaired data. *** significant difference from wildtype (*p* < 0.001).

**Figure 6:**
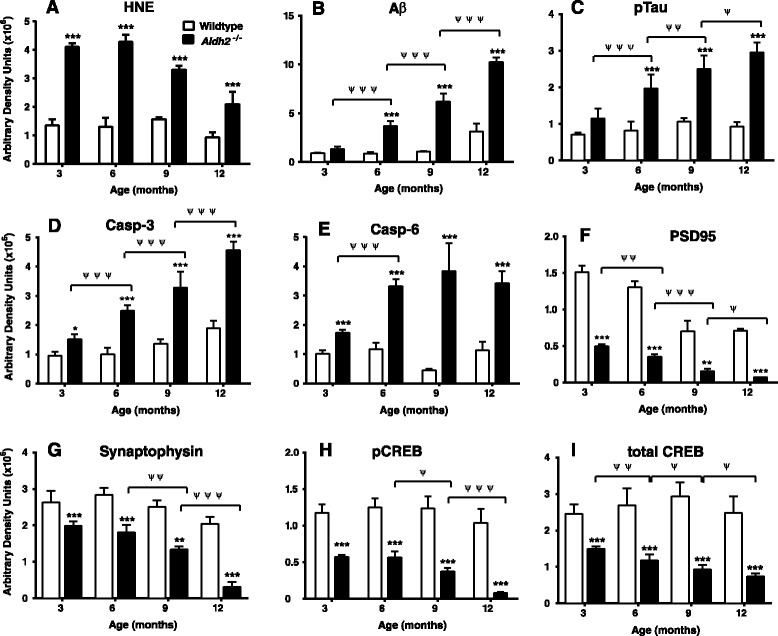
Summary data of immunoblot and dot-blot (HNE) analysis of age-related changes in various AD- and synapse-related markers (panels **A-I**, as indicated) in hippocampal homogenates (30 μg protein) from wildtype or *Aldh2* ^*-/-*^ mice. Immunoreactive bands or spots were quantitated by densitometry. Data are presented as the mean ± SD (n = 4 mice) and were analyzed by a two-way ANOVA with a Bonferroni post-hoc test. *, significant differences from wildtype (*p < 0.05, ***p < 0.001); ^ψ^, significant age-related differences as indicated (^ψ^ *p* < 0.05, ^ψψ^ *p* < 0.001, ^ψψψ^ *p* < 0.001).

**Figure 7:**
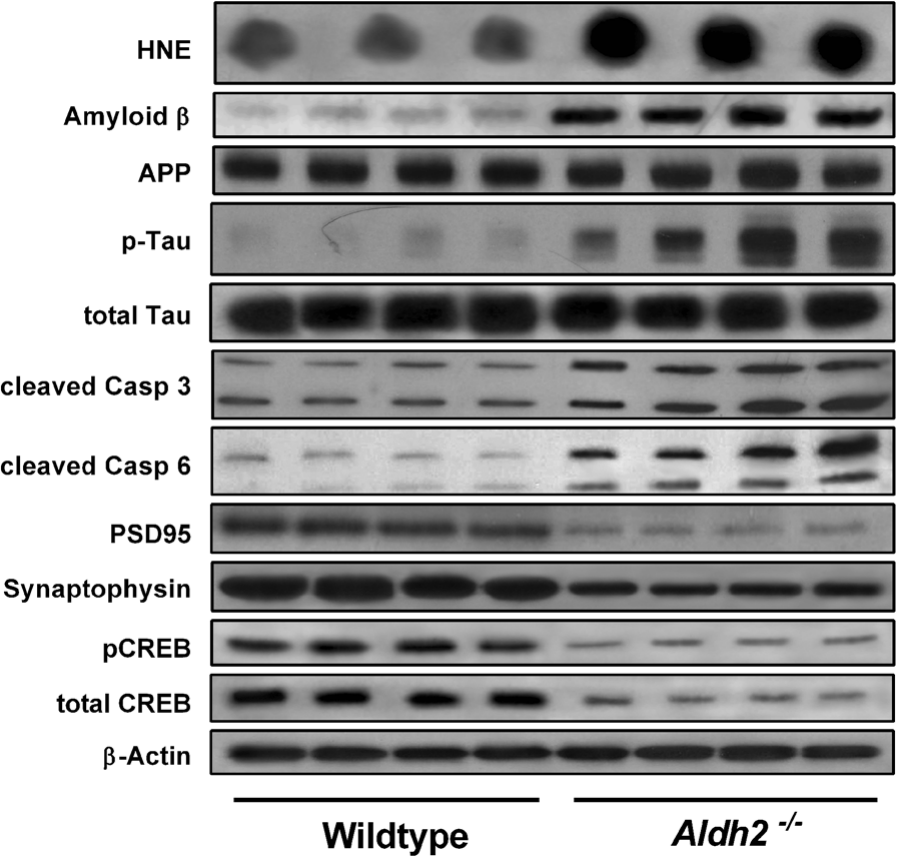
Immunoblot and dot-blot (HNE) analysis of hippocampal homogenates (30 μg protein) from 9 month old wildtype or *Aldh2* ^*-/-*^ mice (n = 4 of each). Note the increase in a number of AD-associated markers in *Aldh2* ^*-/-*^ mice (top part of figure) and the decrease in synaptic markers (bottom part of figure).

**Figure 8:**
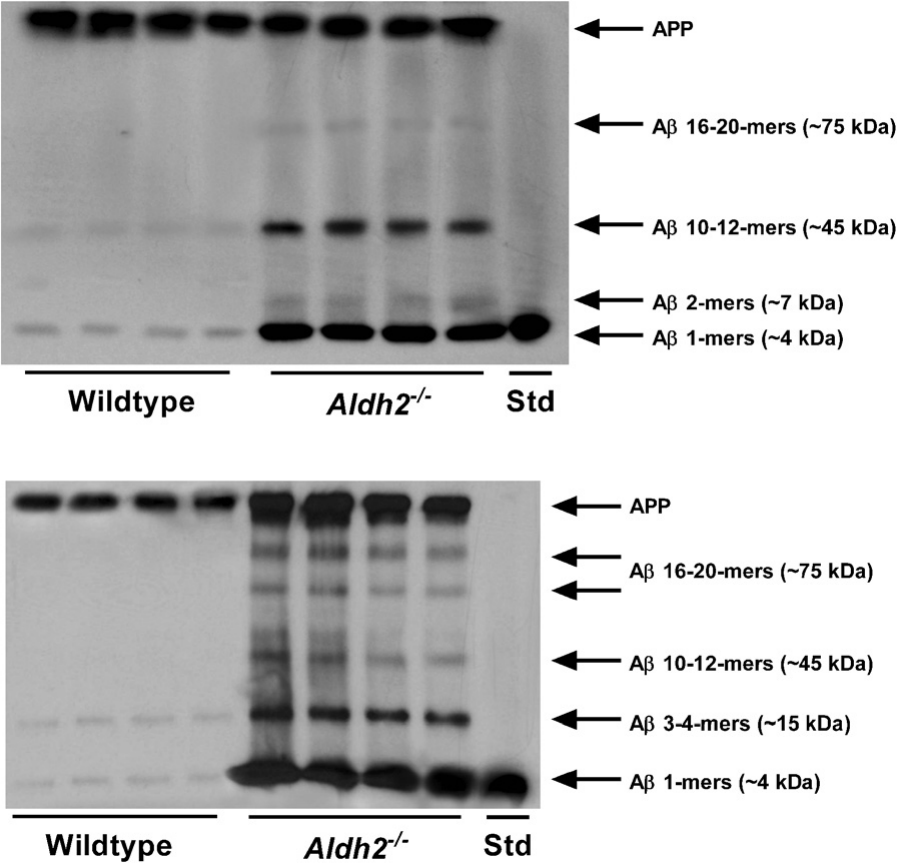
Differential expression of oligomeric forms of Aβ in the hippocampus and cerebral microvasculature of *Aldh2* ^*-/-*^ mice. Immunoblot analysis of hippocampal homogenates (upper panel, 30 μg protein) and cerebral microvessel homogenates (lower panel, 30 μg protein) from 12 month old wildtype or *Aldh2* ^*-/-*^ mice. Samples were added to Laemmli buffer containing 6 M urea, incubated at room temperature for 30 minutes and then resolved on a 15% SDS-PAGE gel. Note the increased expression of APP in the vasculature of *Aldh2* ^*-/-*^ mice in contrast to the equal expression of APP in the hippocampus.

**Figure 9:**
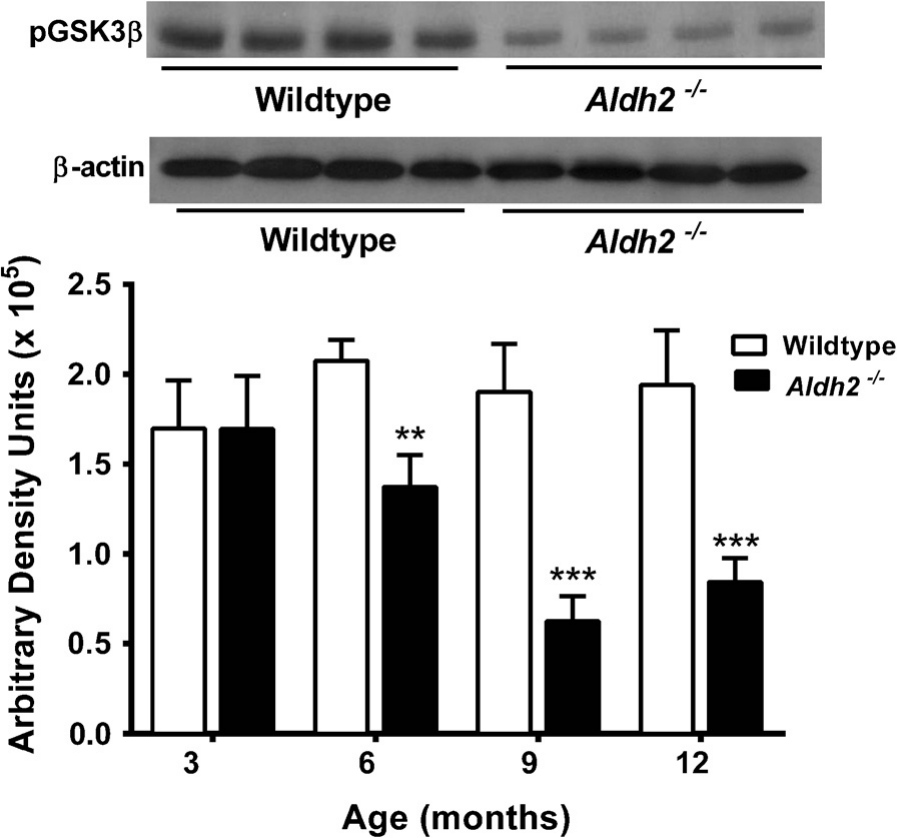
Age-related decreases in phospho-GSK3β (Ser^9^) in hippocampi from *Aldh2* ^*-/-*^ mice. Immunoblot analysis was performed on hippocampal homogenates (30 μg protein) from 3, 6, 9 and 12 month old wildtype or *Aldh2* ^*-/-*^ mice and probed for phosphorylated GSK3β and β-actin. A representative immunoblot is shown from 9 month animals. Immunoreactive bands were quantitated by densitometry. Data are presented as the mean ± SD (n = 4 mice) and were analyzed by a two-way ANOVA with a Bonferroni post-hoc test. *, significant differences from wildtype (**p < 0.01, ***p < 0.001).

**Figure 10:**
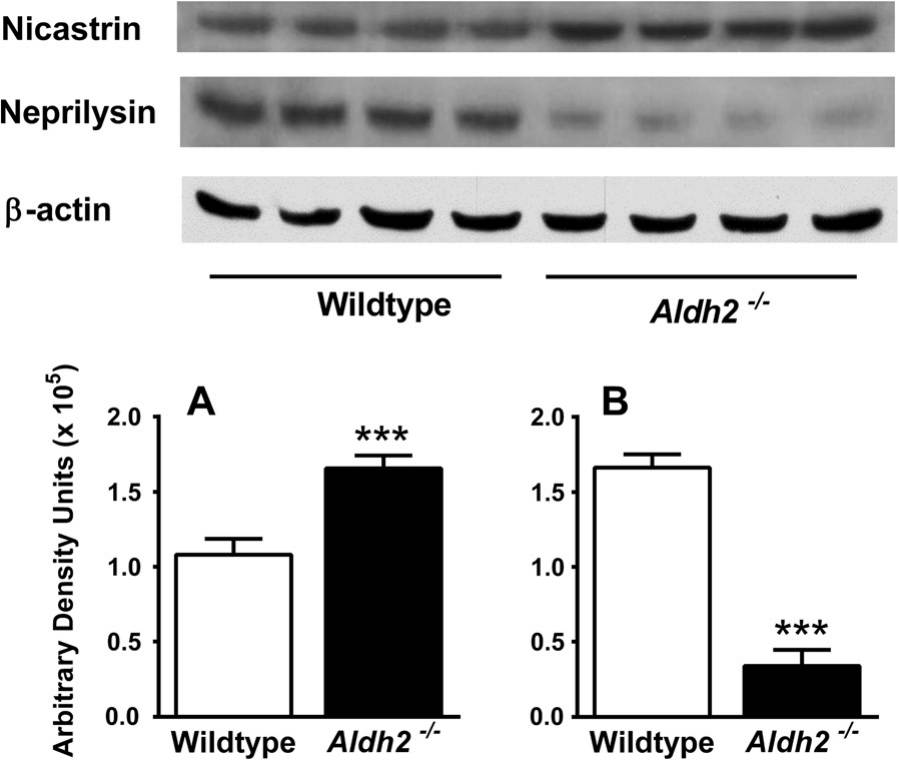
Increased nicastrin and decreased neprilysin expression in hippocampi from 12 month *Aldh2* ^*-/-*^ mice. Immunoblot analysis of hippocampal homogenates (30 μg protein) from 12 month old wildtype and *Aldh2* ^*-/-*^ mice probed for nicastrin **(A)** and neprilysin **(B)**. Immunoreactive bands were quantitated by densitometry. Data are presented as the mean ± SD (n = 4 mice) and were analyzed by Student’s *t*-test for unpaired data. ***, significant difference from wildtype (*p* < 0.001).

HNE adduct formation in *Aldh2*^*-/-*^ mice was characterized by increases in both the density and number of immunoreactive bands (Figure 5). Because of this, we used dot-blot analysis for HNE adduct assessment in subsequent experiments. We followed the age-related changes of a number of relevant markers over a 12 month period at 3 month intervals in hippocampal homogenates from *Aldh2*^*-/-*^ mice (Figure 6; representative immunoblots and HNE dot-blots from 9 month old animals in Figure 7). Marked increases in HNE adduct formation occur as early as 3 months (Figure 6A). Age-related increases in monomeric Aβ and phospho-tau protein (p-tau) were significant at 6 months of age, increasing over the next 6 months (Figure 6B and C). Tau phosphorylation was assessed using the AT-8 antibody which recognizes phosphorylated tau at Ser202, one of the epitopes considered critical for AD progression. Amyloid precursor protein (APP) and total tau expression were unchanged (Figure 7). In addition, a number of oligomeric Aβ (oAβ) species were present in hippocampi from *Aldh2*^*-/-*^ mice (Figure 8). We observed age-related increases in cleaved (activated) caspases 3 and 6 as early as 3 months of age (Figure 6D and E), and age-related decreases in the postsynaptic marker PSD95, the presynaptic marker, synaptophysin, and both total and phosphorylated (Ser133) cAMP-response element binding protein (CREB) (Figure 6F-I). Glycogen synthase kinase 3β (GSK3β) is the most active tau kinase, and *Aldh2*^*-/-*^ mice exhibit age-related decreases in phosphorylation at the major inhibitory site (Ser9) of GSK3β (Figure 9). Increased expression of nicastrin, the γ-secretase substrate receptor, and decreased expression of neprilysin, a major Aβ degrading enzyme, were observed in the hippocampus of older *Aldh2*^*-/-*^ mice (Figure 10).

### CREB and extracellular signal-regulated kinase (ERK) activation in Hippocampal slices

**Figure 11:**
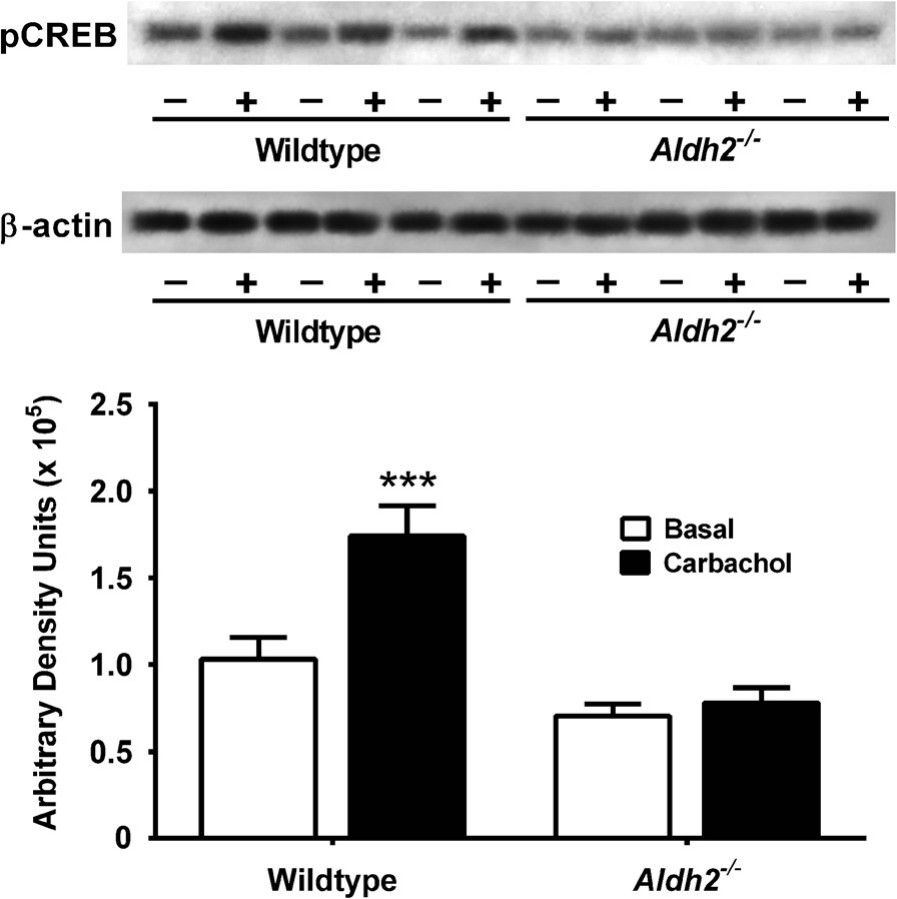
Lack of carbachol-induced phosphorylation of CREB in hippocampi from *Aldh2* ^*-/-*^ mice. Hippocampal slices from 6 month old wildtype and *Aldh2* ^*-/-*^ mice were incubated with 50 μM carbachol (+) or vehicle (-) for 30 min and snap frozen. Immunoblot analysis was performed using 30 μg protein of hippocampal homogenate, and immunoreactive bands were quantitated by densitometry. Data are presented as the mean ± SD (n = 6 mice) and were analyzed by Student’s *t*-test for unpaired data. ***, significant difference from basal (*p* < 0.001).

**Figure 12:**
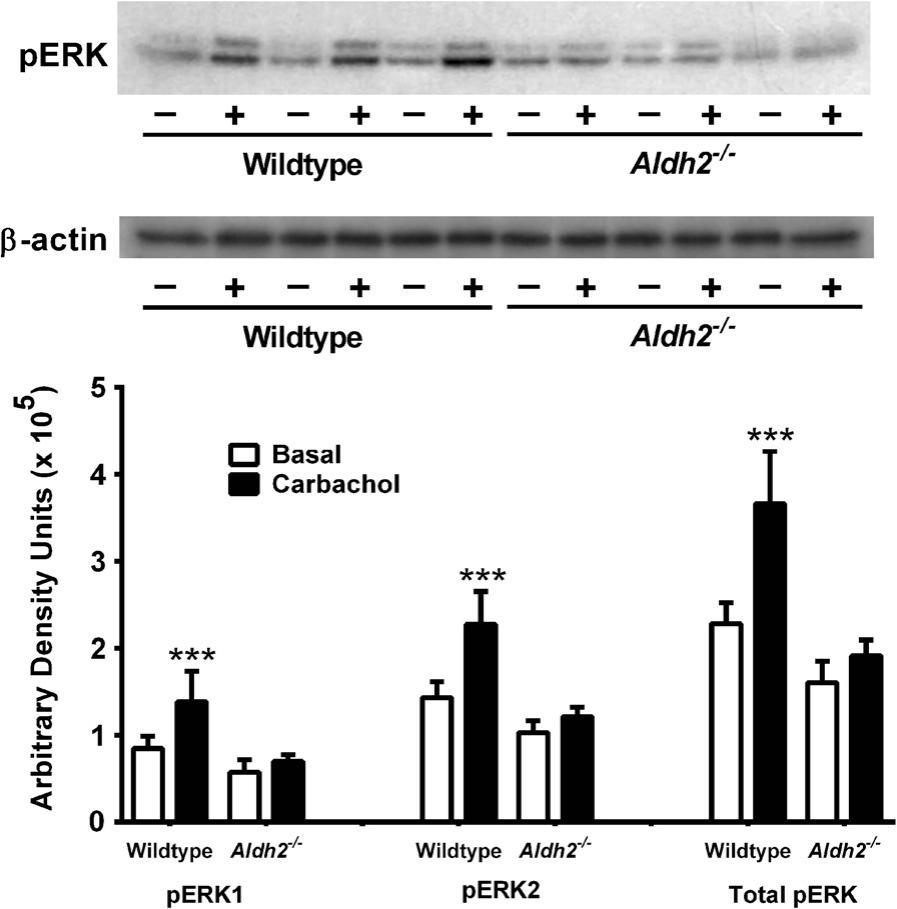
Lack of carbachol-induced phosphorylation of ERK1/2 in hippocampi from *Aldh2* ^*-/-*^ mice. Hippocampal slices from 6 month old wildtype and *Aldh2* ^*-/-*^ mice were incubated with 50 μM carbachol (+) or vehicle (-) for 30 min and snap frozen. Immunoblot analysis was performed using 30 μg protein of hippocampal homogenate, and immunoreactive bands were quantitated by densitometry. Data are presented as the mean ± SD (n = 6 mice) and were analyzed by Student’s *t*-test for unpaired data. ***, significant difference from basal (*p* < 0.001).

In hippocampal slices from 6 month old animals, the increases in pCREB and pERK seen in wildtype mice in response to the cholinergic agonist, carbachol, were absent in *Aldh2*^*-/-*^ mice (Figures 11 and 12), indicating a deficit in cholinergic receptor signaling. In contrast, levels of total CREB and total ERK were unchanged in both wildtype and *Aldh2*^*-/-*^ mice after carbachol treatment (Additional file 2: Figures S2 and Additional file 3: Figure S3).

### Assessment of hippocampal atrophy

The calculated area (mean ± SD) of sections of the hippocampus and overlying neocortex in 33 sections from 6 wildtype mice was 7.4 ± 1.0 mm^3^ and in 27 sections from 7 *Aldh2*^*-/-*^ mice was 6.3 ± 0.6 mm^3^ (*p* < 0.05, Student’s *t*-test for unpaired data).

### Vascular pathologies in *Aldh2*^*-/-*^ mice

**Figure 13:**
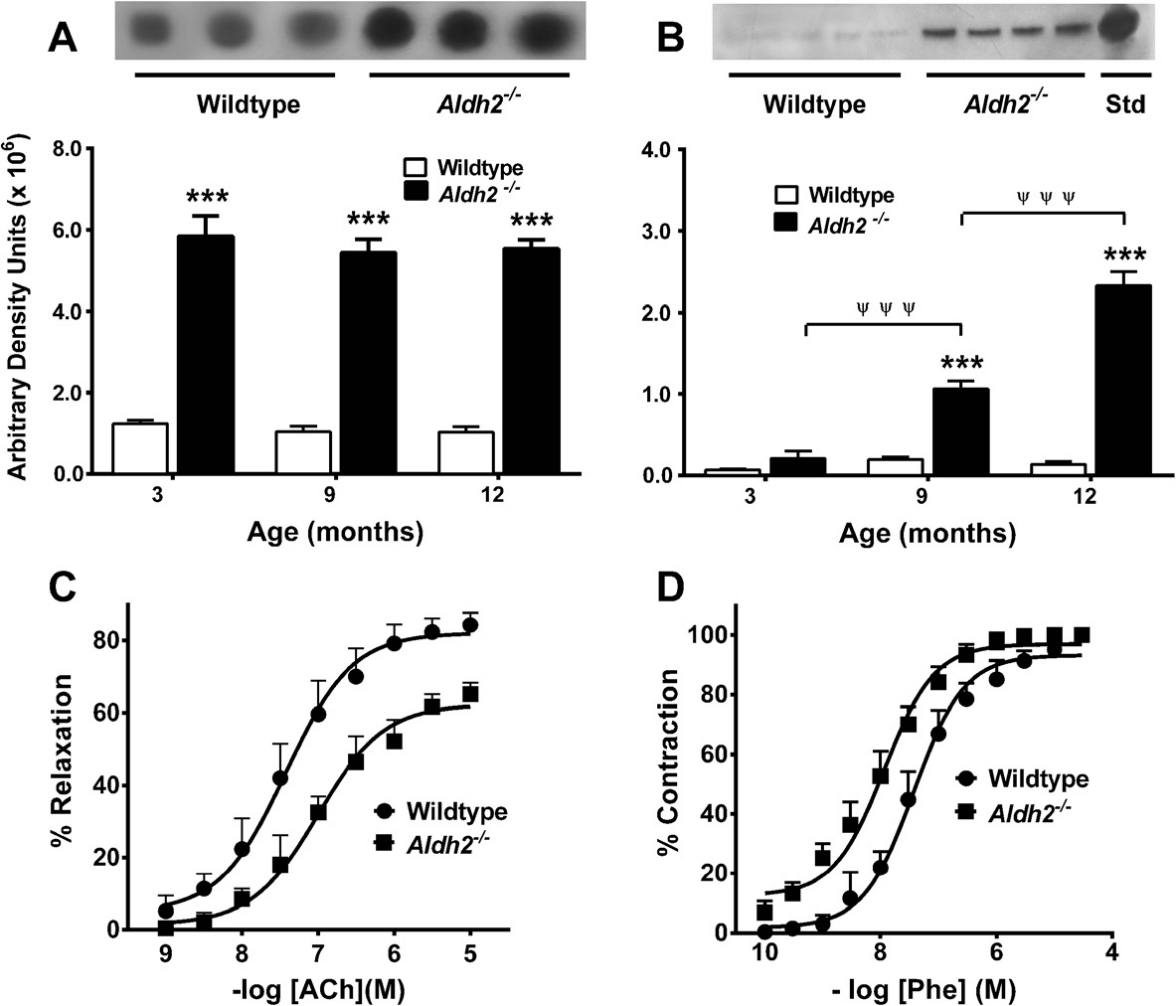
Vascular pathologies in *Aldh2* ^*-/-*^ mice. Cerebral microvessels from *Aldh2* ^*-/-*^ mice exhibit increased HNE adduct formation **(A)** and age-related increases in Aβ formation **(B)**. Representative dot-blots (HNE, 20 μg protein) and immunoblots (Aβ, 30 μg protein) are from 9 month old animals. Immunoreactive spots or bands were quantitated by densitometry. Data are presented as the mean ± SD (n = 4 mice) and were analyzed by a two-way ANOVA with a Bonferroni post-hoc test. ***, significant difference from wildtype (p < 0.001). ^ψ ψ ψ^, significant age-related differences as indicated (p < 0.001). In aortic ring preparations from 12 month old *Aldh2* ^*-/-*^ mice there was a significant decrease in both the potency and maximal relaxation response to the endothelium-dependent vasodilator, acetylcholine (ACh)**(C)**, and a significant increase in the potency of the adrenoceptor agonist, phenylephrine (Phe)** (D)**. Data are presented as the mean ± SD (n = 4 mice) and EC_50_ values for relaxation or contraction were analyzed by Student’s *t*-test for unpaired data.

There was a four-fold increase in HNE adducts in homogenates of mouse cerebral microvessels from 3-12 month old *Aldh2*^*-/-*^ mice (Figure 13A). We observed age-related increases in monomeric Aβ (Figure 13B) as well as a number of oAβ assemblies ranging from 3-4-mers to 20-mers (Figure 8). In addition, endothelial dysfunction and hypercontractility was clearly evident in *Aldh2*^*-/-*^ mice, as indicated by the reduced aortic relaxation responses to acetylcholine (ACh) (Figure 13C), and enhanced contractile responses to the adrenoceptor agonist, phenylephrine (Figure 13D).

## Discussion

The *Aldh2*^-/-^ mouse exhibits progressive, age-related cognitive deficits together with a constellation of AD-associated pathological changes rarely observed in current transgenic AD animal models. These include increases in Aβ, p-tau and activated caspases, synaptic loss, defective CREB signalling, and several vascular pathologies. Although synaptic failure is an early event in AD progression, overt neurodegeneration is either not exhibited or is not an early event in most transgenic AD models [35]. For example, in the 5XFAD mouse, increases in intraneuronal Aβ and Aβ plaques occur at 1.5-2 months of age, whereas the loss of the post-synaptic marker, PSD95, is not evident until animals are 9 months old, and is preceded by the cognitive deficits that occur in 4-5 month old animals [36]. In contrast, in *Aldh2*^-/-^ mice the loss of synaptic markers and the initial deficits in cognitive performance appear together early on in life (at 3 months of age). One advantage of *Aldh2*^-/-^ mice compared to the few other models of sporadic AD that have been described are the availability of appropriate genetic controls (unlike SAMP8 mice [37]). Also, cognitive deficits appear early in life (unlike the degus and rat models [38,39]) but in an age-related manner. A third advantage is that the pathological changes are based upon elevations in a mediator of oxidative stress (HNE) that is considered to be an important contributor to the development of AD pathology. Although there are no rodent models that completely reflect the complexity of human AD, we believe the *Aldh2*^-/-^ mouse represents a valuable addition to currently available transgenic models.

Many studies support the formation of HNE as an early event in the pathogenesis of AD [9-16]. Three detoxification pathways for HNE have been described: conjugation with glutathione by glutathione transferases, reduction by aldo-keto reductases, and oxidation by ALDH2. Studies investigating the expression and activity of ALDH2 and the 3 major aldo-keto reductases responsible for HNE metabolism in the brain found that only ALDH2 expression and activity is increased in AD brains [28,31]. This up-regulation of ALDH2 suggests a protective response to the increases in lipid peroxidation and reactive aldehydes associated with AD pathogenesis. In contrast, glutathione transferase activity is decreased in several brain regions in AD, including the hippocampus [40], and brain levels of several antioxidant enzymes (superoxide dismutase, catalase, glutathione reductase) are also decreased in AD brains [18].

As an oxidative stress-based model of age-dependent cognitive impairment and neurodegeneration, the *Aldh2*^*-/-*^ mice have distinct advantages over mice expressing the Glu504Lys mutation of ALDH2 (ALDH2*2) [33]. Unlike *Aldh2*^*-/-*^ mice, no pathological or cognitive changes were seen in 6 month old ALDH2*2 animals, and memory impairment did not occur until 12 months (vs 3.5-4 months in *Aldh2*^*-/-*^ mice). In 12 month old ALDH2*2 mice there was less than a 2-fold increase in HNE adduct formation (vs 3-fold increases at 3 months in *Aldh2*^*-/-*^ mice). Also <50% of ALDH2*2 mice exhibited neurodegeneration or hyperphosphorylated tau vs increased p-tau in all 6 month old *Aldh2*^*-/-*^ mice, as well as decreased PSD95, synaptophysin and pCREB in all 3 month old *Aldh2*^*-/-*^ mice. Thus *Aldh2*^*-/-*^ mice demonstrate more consistent expression of pathological changes, and at a much younger age, than do transgenic ALDH2*2 mice, highlighting their greater utility, predictability, and cost-effectiveness.

We assessed cognitive performance using three different memory tests. The NOR task is considered a ‘pure’ working memory test, free of any reference memory component. This behavioural test is particularly relevant since visual association tests have been reported to detect, with high specificity, a substantial proportion of AD patients up to a year before diagnosis [42]. The Y-maze task and the version of Morris Water Maze task used here, are tests for spatial working memory and spatial reference memory, respectively. *Aldh2*^-/-^ mice showed a progressive decrease in performance in both the NOR task and the Y-maze tasks, compared to age and sex-matched littermates (Figures 1 and 2). Also, younger (6 month old) animals exhibited decreased performance in the MWM task (Figure 4). Importantly, this progressive decline in memory performance was accompanied by a parallel loss of synaptic markers and of phosphorylated and total CREB (Figure 6F-I).

Age-related increases in monomeric Aβ occur in hippocampi from *Aldh2*^*-/-*^ mice (Figure 6B). In addition, Aβ dimers and oligomeric Aβ (oAβ) of 45 KDa and 75 KDa (corresponding to 10-12-mers and 16-20 mers, respectively) were present in hippocampi from *Aldh2*^*-/-*^ mice (Figure 8). Oligomeric Aβ assemblies are known to induce neurotoxicity *in vitro* and *in vivo* [42-44], and both soluble Aβ and oAβ correlate with disease progression in AD patients [45-47]. Because of sequence differences in the N-terminus of human vs. mouse Aβ, the latter is less prone to aggregate, and endogenous mouse Aβ does not readily form amyloid plaques. Since most studies utilize transgenic mouse models, the formation of mouse oAβ has rarely been investigated or reported. In one study utilizing ApoE4 mice, activation of the amyloid cascade after inhibition of neprilysin did result in the accumulation of prefibrillar oAβ, demonstrating that mouse oAβ can indeed be formed endogenously [48].

Significant increases in HNE adducts of neprilysin [49], one of the principal amyloid-degrading enzymes in the brain, and nicastrin [22], the γ-secretase substrate receptor, occur in AD brain. HNE increases the expression and activity of beta-site APP cleaving enzyme (BACE) and γ-secretase resulting in increased Aβ production [24,50,51]. HNE also forms adducts with neprilysin, resulting in decreased enzymatic activity and Aβ turnover [49,52]. We found increases in nicastrin expression as well as decreases in neprilysin expression in the hippocampus of older *Aldh2*^*-/-*^ mice (Figure 10), which likely contribute to the increased levels of Aβ observed in these mice.

Age-dependent increases in p-tau were observed in *Aldh2*^*-/-*^ mice using the AT8 antibody (Figure 6C). This antibody recognizes phosphorylated tau at Ser202, an epitope associated with intracellular and extracellular filamentous tau [53], and one of the epitopes considered critical for AD progression [54]. Increased tau phosphorylation is seldom observed in transgenic models of AD, and therefore its presence in *Aldh2*^*-/-*^ mice suggests increased HNE contributes to tau pathology. One transgenic mouse model that does exhibit hyperphosphorylated tau and NFTs, the 3xTg-AD mouse, also exhibits oxidative stress, increased lipid peroxidation, and HNE formation as early events, prior to plaque and tangle formation. Furthermore, HNE modifies p-tau, which may play a critical role in the formation of NFTs [55]. GSK3β is the most active tau kinase [54], and has been shown to phosphorylate tau at 42 different sites, 29 of which are found in AD brain [56]. Our data (Figure 9) indicate that *Aldh2*^*-/-*^ mice exhibit age-related decreases in phosphorylation at the major inhibitory site (Ser9) of GSK3β in the hippocampus, suggesting GSK3β activity is increased in *Aldh2*^*-/-*^ mice.

Activation of apoptotic pathways through caspase-mediated cell death contributes to progressive neuronal death in AD [57-60], and we observed age-related increases in cleaved (activated) caspases 3 and 6 as early as 3 months of age in *Aldh2*^*-/-*^ mice (Figure 6D, 6E). Caspase 6 activation has been reported in hippocampus and cerebral cortex of cases of mild cognitive impairment, and of familial and sporadic AD [58,61,62]. In sporadic AD, activated caspase 6 is only present in non-nuclear compartments, and it has been suggested that caspase 6 activation is related to neurodegeneration rather than apoptosis (58). Further, a recent study identified a novel mechanism whereby soluble Aβ-induced caspase 3 activation leads to the downregulation of PSD95 and synaptophysin, resulting in synaptic dysfunction [63].

Synaptic failure and loss of synapses, a hallmark of AD pathology, occurs early in disease progression, prior to plaque and NFT formation and neuronal death. Synaptic loss is a key event in early cognitive decline [64] and a highly relevant correlate of cognitive deficits in AD [65]. As an index of neurodegeneration and synaptic loss, we assessed hippocampal levels of the postsynaptic protein, PSD95, a major scaffolding molecule localized at the postsynaptic density of excitatory glutamatergic synapses, and of synaptophysin, a synaptic vesicle protein and presynaptic marker. There were marked, age-related decreases in both PSD95 and synaptophysin beginning at 3 months (Figure 6F, 6G), suggesting that hippocampal synaptic loss in these mice is an early event that occurs concomitant with the development of cognitive deficits. There was also evidence of brain atrophy in these mice; measurements of the area of hippocampal formation and overlying neocortex indicated a 15% reduction in area in *Aldh2*^*-/-*^ mice.

The induction of structural changes at synapses requires activation of gene networks and transcription factors such as CREB, and an association between activity-dependent gene expression and CREB activation is well established [66,67]. Activation of CREB by phosphorylation at Ser133 is critical for memory formation and synaptic strengthening [68] and mediates long term potentiation (LTP) by acting upon downstream genes involved in synaptic formation and maintenance, neuronal plasticity and neurogenesis [69]. Decreased levels of pCREB are seen in the hippocampus of AD brain [70], and molecular network analysis suggests a central role of aberrant CREB-mediated gene regulation in AD [71]. Restoration of CREB signalling restores LTP and regulates gene products, notably BDNF, leading to neuroprotection and restoration of neuronal function. Studies have also reported reciprocal regulation of amyloidogenesis by CREB and direct dysregulation of CREB activation by Aβ [72-75]. We observed marked decreases in basal pCREB and total CREB levels in *Aldh2*^*-/-*^ mice (Figure 6H, 6I). Furthermore, the increases in pCREB and pERK seen in hippocampal slices of the wildtype mice in response to cholinergic receptor activation by carbachol are absent in *Aldh2*^*-/-*^ mice (Figures 11 and 12), indicating a deficit in cholinergic receptor signaling.

In addition to the observed AD-like pathologies in the brains of *Aldh2*^*-/-*^ mice, we found significant vascular alterations in cerebral microvessels (CMVs) of these mice. Marked increases in HNE adducts and age-related increases in monomeric Aβ were found in CMVs from *Aldh2*^*-/-*^ mice compared to wildtype (Figure 13A, 13B), as well as a number of oAβ assemblies ranging from 3-4-mers to 20-mers (Figure 8). Cerebral amyloid angiopathy (CAA) is characterized by the accumulation of Aβ in CMVs. It is a common pathological feature in AD occurring in 60-90% of AD patients [76] and also occurs in some transgenic animal models [77]. The presence of CAA significantly worsens cognitive performance in the early stages of AD [78]. Endothelial dysfunction and arterial hypercontractility have been associated with the AD phenotype [79,80], and the degree of endothelial dysfunction correlates with the AD severity [79]. Endothelial dysfunction and hypercontractility were clearly evident in *Aldh2*^*-/-*^ mice, as indicated by the reduced aortic relaxation responses to acetylcholine that are dependent on endothelial NO release, and by enhanced contractile responses to the adrenoceptor agonist, phenylephrine (Figure 13C, 13D). Thus, this mouse model incorporates both neural and vascular pathological changes associated with AD.

## Conclusions

We have characterized a novel oxidative stress-based mouse model of cognitive impairment with AD-like biochemical and structural pathologies based on increased HNE formation due to the genetic deletion of ALDH2. These mice exhibit age-dependent cognitive impairment, synaptic loss, neurodegeneration, altered CREB signalling, increased Aβ, hyperphosphorylated tau, activated caspases, and vascular pathologies. The *Aldh2*^*-/-*^ animal model exhibits elevated Aβ, and more importantly, elevated p-tau and neurodegeneration, significant human pathologies rarely seen together in current transgenic models. We believe that this new model of age-related cognitive impairment is a valuable addition to currently available transgenic models and will provide new insight into the pathogenesis and molecular/cellular mechanisms driving neurodegenerative diseases of aging such as AD. It may be useful for delineating the chronology of appearance of AD biomarkers, identifying targets for early intervention, and assessing of the efficacy of therapeutic agents for improving memory and for slowing, preventing, or reversing the synaptic failure and neurodegeneration associated with AD.

## Methods

### Generation of *Aldh2*^*-/-*^ mice

All procedures for animal experimentation were undertaken in accordance with the principles and guidelines of the Canadian Council on Animal Care and were approved by the Queen’s University Animal Care Committee. Animals were maintained under a 12 h light/dark cycle, with free access to food and water. The *Aldh2*^*-/-*^ mice have a C57BL/6 background and were generated by gene targeting knockout as previously described [81] and kindly provided by Dr. T. Kawamoto (University of Occupational and Environmental Health, Kitakyushu, Japan). Wildtype male C57BL/6 mice (20 -30 g) were obtained from Jackson Laboratory, (Bar Harbor ME) and backcrossed with *Aldh2*^*-/-*^ mice for more than 10 generations. Wildtype and *Aldh2*^*-/-*^ cohorts were generated by mating heterozygotes, and genotyping of the progeny by PCR analysis of genomic DNA extracted from tail tips or ear punches using the primers as reported [82].

### Behavioural analyses

*Open-field Novel Object Recognition (NOR) task.* The NOR task consisted of three consecutive days of testing per trial; habituation, training with two identical objects, and testing with one familiar and one novel object. On the testing day, animals were allowed to explore the objects until they accumulated 30 seconds of total object exploration time (exploration was recorded when the nose of the mouse was within approximately 1cm of the object). This was done rather than using a set exposure time in the test environment in order to account for any variability in movement and exploration that may occur between mice. Two measures of behaviour were assessed: frequency of visits to the objects, and the time spent exploring each object. From the latter measure the discrimination index (difference in time exploring the novel and familiar object, divided by total exploration time) and the ratio of time spent with the novel object in relation to the familiar object were calculated. *Y-maze task*. Mice were placed in the center of the maze and allowed to explore the three maze arms freely for 10 min. The maze was surrounded by distinct spatial cues so that the mice could distinguish between arms. The spontaneous alternation rate was calculated as the total triads containing entries into each of the three arms without repeated entry into a previously visited arm, divided by the total number of arm entries. *Morris Water Maze task*. The maze consisted of a circular pool (1.2m in diameter, [83]) filled with water (approximately 23°C) made opaque by non-toxic white paint. A circular platform (15cm in diameter) was submerged approximately 1cm below the surface in the northeast (NE) quadrant of the maze and, thus, hidden from view. The escape latency (time to reach the hidden platform) was determined in in a 3 day cued trial block (4 trials per day) followed by a 5 day hidden trial block (6 trials per day) [84]. For cued platform training, a black-and-white striped pole (2.2 cm diameter and 15 cm height) was attached to the center of the platform. Each mouse was allowed 60 seconds to locate the platform per trial. Mice that could not find the platform within 60 seconds were gently guided towards it. Day 9 was a 60 second probe trial, in which the time spent in each quadrant and number of platform crosses were recorded. *Balance beam task.* This task consisted of three consecutive days of testing on a 1 meter long beam with a flat surface (1.8 cm wide) resting about 50cm above the floor on two supports. An empty cage with nesting material served as a finish point at the end of the beam to attract the mouse. Day 1 and day 2 were training days in which the mouse was encouraged to cross the beam 3 times with a 10 minute break between each trial. On day 3, the mice were again given 3 trials, and the two best times required to cross the beam and enter the finish box were averaged [85].

### Immunoblot and dot-blot analysis

Mouse hippocampi were isolated and homogenized in lysis buffer (25 mM HEPES pH 7.0, 1 mM EDTA, 1 mM EGTA, 1% Triton X-100, 0.1% SDS, protease inhibitors and phosphatase inhibitors (Roche Diagnostics, Mannheim, Germany)) and centrifuged at 10000*g* for 10 minutes. Proteins in the supernatant fraction were separated by SDS**-**PAGE on 10-15% gels (depending on protein of interest) and transferred electrophoretically to PVDF membranes. For the immunoblot analysis of HNE adducts in Figure 5, proteins were resolved on a 10% SDS-PAGE gel under non-reducing conditions. In all other experiments, HNE adducts were assessed by dot-blot analysis, in which homogenates were spotted onto a PVDF membrane and dried before probing with antibody. For immunoblot analysis of Aβ, samples were added to Laemmli buffer containing 6 M urea and incubated at room temperature for 30 minutes prior to electrophoresis. Immunoblots or HNE dot-blots were probed with primary antibody followed by secondary antibody, and immunoreactive bands or spots visualized by enhanced chemiluminescence. Immunoreactive bands or spots were quantified by optical densitometry using ImageJ software (version 1.43). Blots were stripped and reprobed for β-actin to confirm equal loading. The following antibodies were used: mouse monoclonal to Aβ (W0-2, Millipore, recognizes residues 4-10 of Aβ), mouse monoclonal to APP (2C11, Millipore), rabbit polyclonal to 4-hydroxynonenal (HNE11-S, Alpha Diagnostics International), mouse monoclonal to PHF-tau (AT8, Pierce Thermo Scientific), mouse monoclonal to total tau (ab64193, Abcam), rabbit polyclonal to caspase-3 (ab90437, Abcam), rabbit polyclonal to caspase-6 (ab52295, Abcam), mouse monoclonal to PSD95 (Pierce Thermo Scientific), mouse monoclonal to synaptophysin (ab8049, Abcam), rabbit polyclonal to phospho-CREB (Ser133) (06-519, Millipore), rabbit polyclonal to total CREB (06-863, Millipore), mouse monoclonal to phospho-ERK1/2 (05-481, Millipore), rabbit polyclonal to ERK1/2 (06-182, Millipore), rabbit polyclonal to phospho-GSK3β (Ser9)(ab131097, Abcam), rabbit polyclonal to nicastrin (ab45425, Abcam) mouse monoclonal to neprilysin (ab951, Abcam) and mouse monoclonal to β-actin (Sigma).

### CREB and ERK activation in hippocampal slices

Mouse hippocampal mini-slices containing the CA1 region were prepared as we have described for rat [86]. After a 3 h equilibration in Krebs’ buffer, slices were exposed to the cholinergic receptor agonist, carbachol (50 μM) for 30 min, conditions in which we have observed increases in pERK in rat hippocampal mini-slices [86]. After incubation, slices were homogenized in lysis buffer (50mM TRIS, pH 7.4, 50mM sodium pyrophosphate, 1mM EDTA, 1% Triton X-100, 1mM dithiothreitol, 1mM PMSF, 1mM NaF, 1mM NaVO4 and protease and phosphatase inhibitors), and total and phosphorylated ERK and CREB assessed by immunoblot analysis.

### Preparation of cerebral microvessels

Mouse cerebral microvessels were prepared using a modified mechanical dispersion and filtration technique as previously described [87]. Briefly, cortices were isolated, cleaned, and carefully homogenized by hand using a Dounce homogenizer in 5-fold excess volume of phosphate-buffered saline with protease and phosphatase inhibitors (Roche Diagnostics, Mannheim, Germany). Dextran was added to a final concentration of 15% and the homogenate was centrifuged (6200g for 30 minutes at 4°C). The pellet was resuspended in the PBS solution and passed through a 40 μm mesh filter to capture microvessels. The microvessels were homogenized in lysis buffer (25 mM HEPES pH 7.0, 1 mM EDTA, 1 mM EGTA, 1% Triton X-100, 0.1% SDS, protease inhibitors and phosphatase inhibitors (Roche Diagnostics, Mannheim, Germany)) and used for immunoblot and dot-blot analysis.

### Assessment of hippocampal atrophy

For assessment of hippocampal atrophy, measurements were made of coronal sections cut at -2.30 to -2.10 Bregma. The neocortical surface and base of the hippocampal formation were outlined and connected by 2 lines bounding the hippocampal formation and meeting the neocortical surface at ninety degrees. The area bounded by these perimeter lines was calculated using the imaging software associated with the microscope. 

### Isolated blood vessel preparations

Isolated rings of aorta (2-3 mm) were prepared for isometric tension measurements and were equilibrated for 1 h at an optimal resting tension of 5 mN. Cumulative concentration-response curves for phenylephrine (0.1 nM-30 μM) were then obtained. After washout, aortic rings were contracted submaximally with phenylephrine (0.2–5 μM), and after the induced tone had stabilized, cumulative concentration-response curves were obtained for acetylcholine (ACh) (1 nM–10 μM).

### Data analysis

Data is expressed as the mean ± SD and was analyzed by one-, two- or three-way analysis of variance with Bonferroni’s post-hoc test, and/or a two-tailed Student’s *t* test for unpaired data, as indicated. A *p*-value of 0.05 or less was considered statistically significant. Relaxation responses to ACh were measured as the percentage decrease in phenylephrine-induced tone. EC_50_ values for relaxation and contraction were determined from the concentration-response curves using a sigmoidal dose–response curve-fitting algorithm. Due to inhomogeneity of variance, statistical analysis for the blood vessel experiments was performed using logarithmically transformed data.

## Additional files

Additional file 1: Figure S1. Performance in the Balance Beam Test is unaltered in *Aldh2* ^*-/-*^ mice. Performance on the balance beam was quantified by measuring the total time taken for the mouse to transverse the beam. Differences in performance were not observed in either 2-3 month old (A) or 5-6 month old (B) animals. Data are presented as the mean ± SD (wildtype n = 15, *Aldh2* ^*-/-*^ n = 20) and were analyzed using Student’s *t*-test for unpaired data (p > 0.05). Additional file 2: Figure S2. Lack of carbachol-induced changes in total CREB in hippocampi from wildtype or *Aldh2* ^*-/-*^ mice. Hippocampal slices from 6 month old wildtype and *Aldh2* ^*-/-*^ mice were incubated with 50 μM carbachol (+) or vehicle (-) for 30 min and snap frozen. Immunoblot analysis was performed using 30 μg protein of hippocampal homogenate, and immunoreactive bands were quantitated by densitometry. Data are presented as the mean ± SD (n = 6 mice) and were analyzed by Student’s *t*-test for unpaired data (p > 0.05). Additional file 3: Figure S3. Lack of carbachol-induced changes in total ERK in hippocampi from wildtype or *Aldh2* ^*-/-*^ mice. Hippocampal slices from 6 month old wildtype and *Aldh2* ^*-/-*^ mice were incubated with 50 μM carbachol (+) or vehicle (-) for 30 min and snap frozen. Immunoblot analysis was performed using 30 μg protein of hippocampal homogenate, and immunoreactive bands were quantitated by densitometry. Data are presented as the mean ± SD (n = 6 mice) and were analyzed by Student’s *t*-test for unpaired data (p > 0.05).

## Abbreviations

Aβ: Amyloid beta peptide
ACh: Acetylcholine
AD: Alzheimer’s disease
ALDH2: Aldehyde dehydrogenase 2
APP: Amyloid precursor protein
BACE: Beta-site APP cleaving enzyme
CAA: Cerebral amyloid angiopathy
CMV: Cerebral microvessels
CREB: Cyclic AMP response element binding protein
ERK: Extracellular signal-regulated kinase
GSK3β: Glycogen synthase kinase 3β
HNE: 4-hydroxynonenal
NFT: Neurofibrillary tangle
NOR: Novel object recognition
