# Supplementary figures and images for: Characterization of Aldh2-/- mice as an age-related model of cognitive impairment and Alzheimer’s disease
Source: Mol Brain. 2015 Apr 25;8:27. doi: 10.1186/s13041-015-0117-y (PMC4409701; doi:10.1186/s13041-015-0117-y)

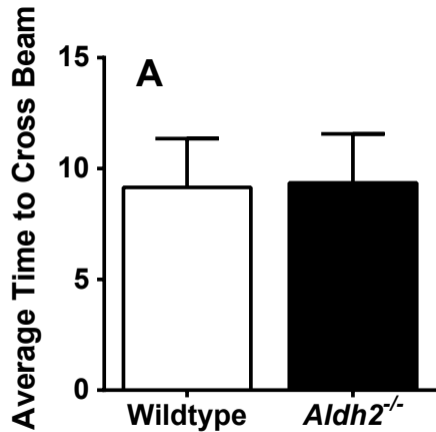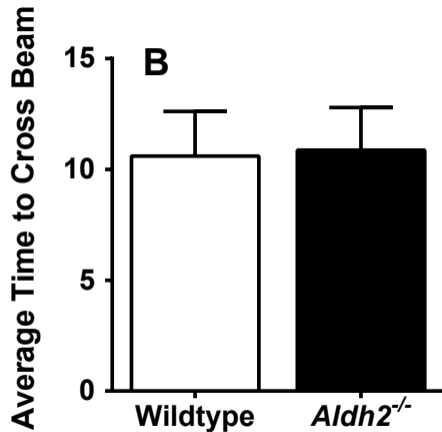

Supplement: Additional file 1: Figure S1. — Performance in the Balance Beam Test is unaltered in Aldh2 -/- mice. Performance on the balance beam was quantified by measuring the total time taken for the mouse to transverse the beam. Differences in performance were not observed in either 2-3 month old (A) or 5-6 month old (B) animals. Data are presented as the mean ± SD (wildtype n = 15, Aldh2 -/- n = 20) and were analyzed using Student’s t-test for unpaired data (p > 0.05). [file 13041_2015_117_MOESM1_ESM.pdf]

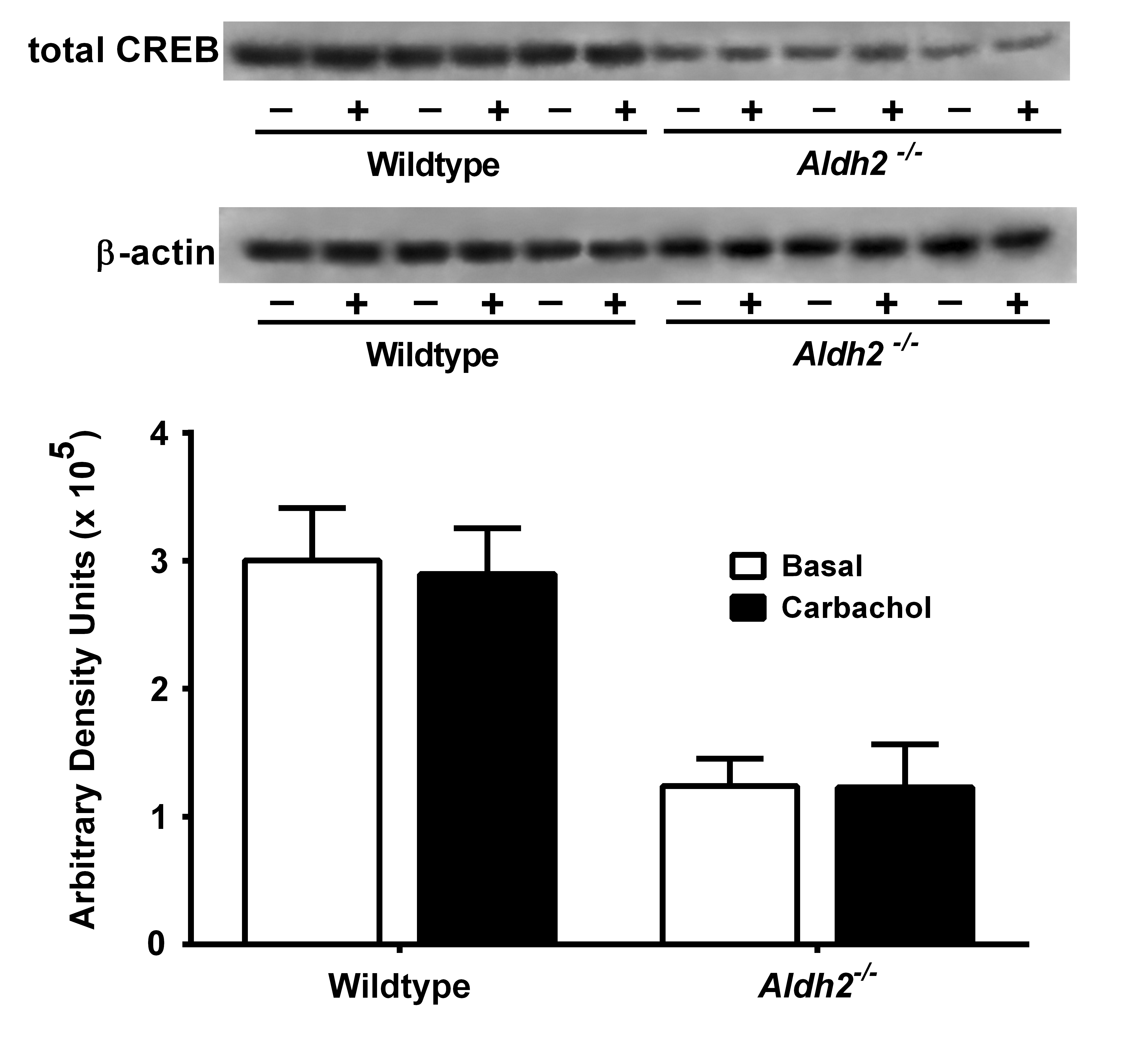

Supplement: Additional file 2: Figure S2. — Lack of carbachol-induced changes in total CREB in hippocampi from wildtype or Aldh2 -/- mice. Hippocampal slices from 6 month old wildtype and Aldh2 -/- mice were incubated with 50 μM carbachol (+) or vehicle (-) for 30 min and snap frozen. Immunoblot analysis was performed using 30 μg protein of hippocampal homogenate, and immunoreactive bands were quantitated by densitometry. Data are presented as the mean ± SD (n = 6 mice) and were analyzed by Student’s t-test for unpaired data (p > 0.05). [file 13041_2015_117_MOESM2_ESM.png]

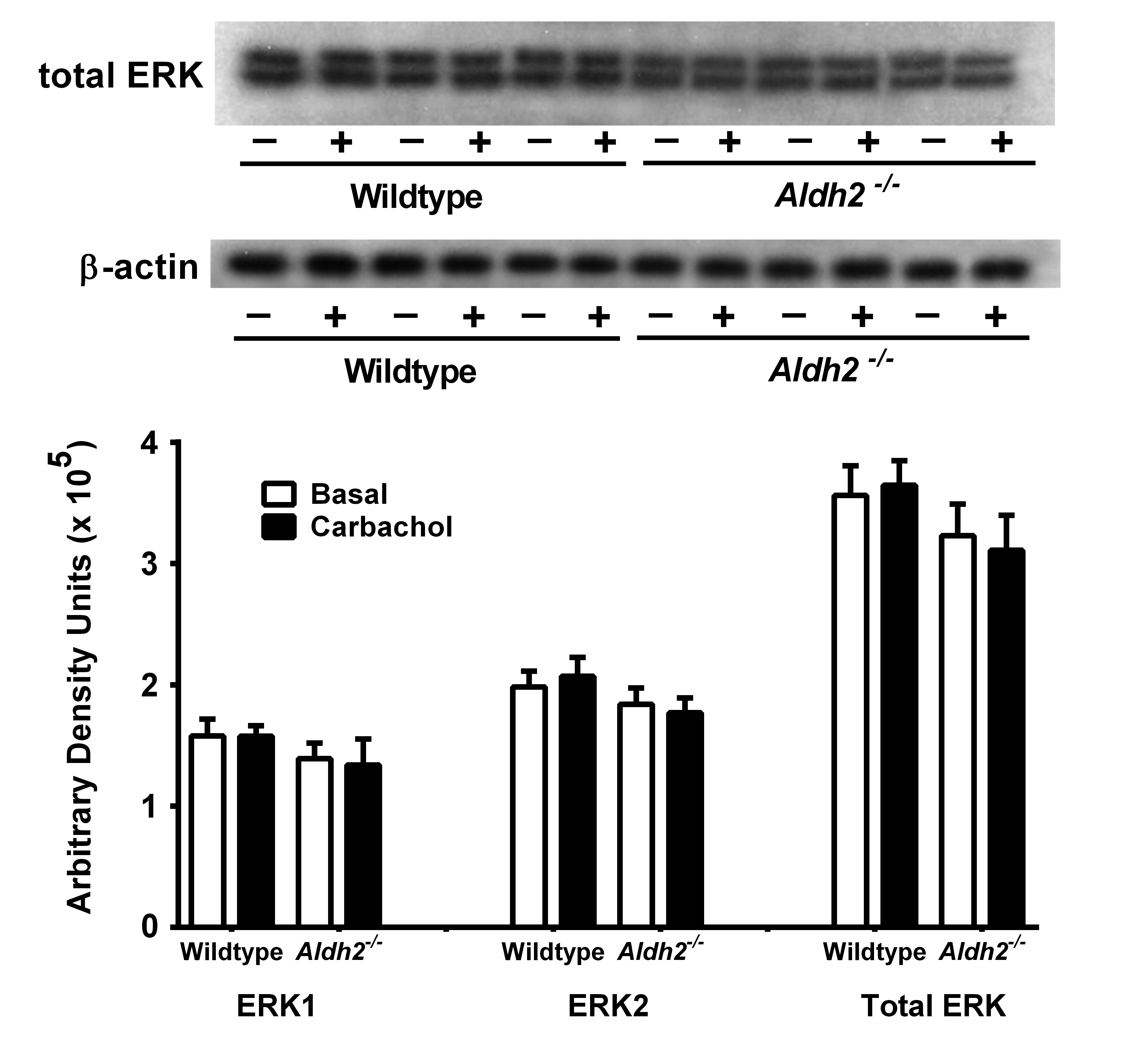

Supplement: Additional file 3: Figure S3. — Lack of carbachol-induced changes in total ERK in hippocampi from wildtype or Aldh2 -/- mice. Hippocampal slices from 6 month old wildtype and Aldh2 -/- mice were incubated with 50 μM carbachol (+) or vehicle (-) for 30 min and snap frozen. Immunoblot analysis was performed using 30 μg protein of hippocampal homogenate, and immunoreactive bands were quantitated by densitometry. Data are presented as the mean ± SD (n = 6 mice) and were analyzed by Student’s t-test for unpaired data (p > 0.05). [file 13041_2015_117_MOESM3_ESM.png]
